# The Relative Reinforcing Value of Menthol Among Young Adult Cigarette Smokers: Results From a Behavioral Choice Task

**DOI:** 10.1093/ntr/ntae186

**Published:** 2024-09-18

**Authors:** Amy M Cohn, Hoda Elmasry, Rebecca Ashare, Wallace Pickworth, James G Murphy, Andrea C Villanti, Donald Hedeker, Delaney Dunn, Riley Wyatt, Taylor Niznik, Whitney D Margaritis, Michael A Smith, Sarah J Ehlke, Janet Audrain-McGovern

**Affiliations:** Department of Pediatrics, University of Oklahoma Health Sciences Center, Oklahoma City, OK, USA; TSET Health Promotion Research Center, Stephenson Cancer Center, University of Oklahoma Health Sciences Center, Oklahoma City, OK, USA; Department of Pediatrics, University of Oklahoma Health Sciences Center, Oklahoma City, OK, USA; Independent Contractor, Ashburn, VA, USA; Department of Psychology, University at Buffalo, Buffalo, NY, USA; Battelle Memorial Institute, Baltimore, MD, USA; Department of Psychology, University of Memphis, Memphis, TN, USA; Rutgers Institute for Nicotine & Tobacco Studies, Rutgers Biomedical and Health Sciences, New Brunswick, NJ, USA; The Department of Public Health Sciences, University of Chicago, Chicago, IL, USA; TSET Health Promotion Research Center, Stephenson Cancer Center, University of Oklahoma Health Sciences Center, Oklahoma City, OK, USA; TSET Health Promotion Research Center, Stephenson Cancer Center, University of Oklahoma Health Sciences Center, Oklahoma City, OK, USA; TSET Health Promotion Research Center, Stephenson Cancer Center, University of Oklahoma Health Sciences Center, Oklahoma City, OK, USA; TSET Health Promotion Research Center, Stephenson Cancer Center, University of Oklahoma Health Sciences Center, Oklahoma City, OK, USA; TSET Health Promotion Research Center, Stephenson Cancer Center, University of Oklahoma Health Sciences Center, Oklahoma City, OK, USA; TSET Health Promotion Research Center, Stephenson Cancer Center, University of Oklahoma Health Sciences Center, Oklahoma City, OK, USA; Department of Psychiatry, Perelman School of Medicine of the University of Pennsylvania, Philadelphia, PA, USA

## Abstract

**Introduction:**

Menthol cigarettes are associated with experimentation and progression to regular use. Although reinforcement processes likely underlie menthol’s appeal, the reinforcing value of menthol cigarettes remains unknown.

**Aims and Methods:**

This study examined the relative reinforcing value (RRV) of menthol versus nonmenthol cigarettes in young adult menthol (*n* = 54) and nonmenthol (*n* = 53) smokers, and differences in menthol’s RRV by race, ethnicity, and sexual orientation. Overnight abstinent participants completed a choice task assessing willingness to “work” to click targets on a computer screen to earn menthol or nonmenthol cigarette puffs. A progressive ratio schedule was used where the menthol target had to be clicked progressively more times, over 10 trials, to earn a menthol cigarette puff, while clicks for the nonmenthol target were fixed across trials. RRV for menthol was defined by the breakpoint, or the highest trial (out of to 10) completed for a menthol cigarette puff. Number of clicks for menthol and nonmenthol puffs were also examined.

**Results:**

Menthol smokers worked harder for menthol versus nonmenthol cigarette puffs (breakpoint = 9.17; ~1236 clicks vs. 24 clicks). Breakpoint was higher among Hispanic (6.49) versus NH White (4.83) and NH non-White smokers (4.43). In exploratory analyses of interactions of menthol preference with race and ethnicity, nonmenthol Hispanic smokers worked harder for menthol cigarette puffs versus NH non-White and NH White nonmenthol smokers.

**Conclusions:**

Menthol cigarettes are highly reinforcing for young adult menthol and Hispanic smokers. A menthol ban may reduce addiction risk among younger individuals and some minoritized groups of smokers.

**Implications:**

This study provides evidence of the greater relative reinforcing value of menthol compared to nonmenthol cigarettes among young adults who had a cigarette flavor preference, suggesting increased addiction risk of menthol cigarettes. Young adult menthol smokers and Hispanic (vs. non-Hispanic) smokers worked harder to earn menthol (vs. nonmenthol) cigarette puffs. Findings add to the evidence base supporting the U.S. Food and Drug Administration’s (FDA) intent to ban menthol in cigarettes. Further, prevention messaging campaigns and cessation programs should take into account the reinforcing value of menthol in cigarettes, especially in vulnerable and at-risk populations.

## Introduction

Menthol smoking has increased in young adult smokers, while nonmenthol smoking has decreased.^1^ Most new smokers initiate with a menthol cigarette and menthol is one of the most popular flavored tobacco products used by young adults.^2,3^ Experimentation with menthol cigarettes is associated with smoking progression, nicotine dependence,^4,5^ and worse cessation outcomes.^6^ According to U.S. national studies, menthol cigarettes are disproportionately used by populations with tobacco use disparities, including Black and Hispanic individuals^1,7^ and those identifying as a sexual minority (e.g., lesbian, gay, bisexual, queer, questioning, or some other nonheterosexual identity).^1^ In 2022, the FDA proposed to ban menthol cigarettes to improve public health, prevent initiation, and reduce tobacco-related disparities.

According to the Positive Reinforcement Model of Addiction, menthol use is maintained by positive, pleasurable, and other hedonic responses to smoking.^8,9^ Menthol adds a minty flavor to tobacco and has been hypothesized to mask the harshness of inhaled cigarette smoke,^9^ increasing the pleasantness and palatability of smoking.^10–13^ Menthol’s pleasurable taste and other positive sensory effects (e.g., throat grab) may contribute to a more positive initial smoking experience,^12^ a potential mechanism linking uptake with continued smoking.^2,12^ Menthol’s positive sensory effects may also contribute to the misperception that menthol cigarettes are less harmful and addictive than nonmenthol cigarettes,^14^ thereby increasing intentions to use menthol cigarettes,^15^ lowering desire to quit,^16^ or enhancing nicotine exposure through more intense smoking (as evidenced in some laboratory studies).^10,17^ Over time, the conditioned reinforcing aspects of menthol flavoring on smoking could strengthen the learned association between smoking and reward, beyond nicotine alone.^8,18^ Menthol’s appeal may be further magnified in groups that are targeted by tobacco company marketing, including Black, Hispanic, and sexual minority individuals.^14,19,20^ Collectively, menthol flavoring may increase the reinforcing effects of smoking through a variety of factors^21^ (e.g., positive subjective effects, lower perceptions of harm, tobacco company targeting, etc.), making it easier to start smoking and harder to quit.

Behavioral economic measurement paradigms can assess the tobacco-related decisional processes affected by product descriptors, sensory experiences, and marketing, all factors on which tobacco companies capitalize.^22^ The term relative reinforcing value (RRV) is used in the context of behavioral economic research to describe the strength of reinforcement of one commodity (e.g., menthol cigarette) compared to available alternatives^23^ (e.g., nonmenthol cigarette). Behavioral economic theory posits that appeal for drugs, or behavioral economic demand, is measured by examining the degree of resource allocation for a drug, and the extent to which consumption is sensitive to contingencies, such as price, availability, or degree of effort to obtain that drug.^24^ It can also be operationalized as the distribution of time, effort, or behavior among alternative sources of reinforcement based on the perceived value of a commodity.^24–27^ In this way, the more resources (e.g., time, money, effort) one is willing to expend for one commodity (versus an alternative) is indicative of greater RRV for that particular commodity.^28–30^

Only a few studies have explored the RRV of menthol versus nonmenthol cigarettes using behavioral economic approaches. Most have been conducted in older adult samples (e.g., average aged mid-forties), and yield complementary results.^10,13,30–34^ Two survey studies employed cigarette purchase tasks, gauging participants’ hypothetical willingness to buy menthol or nonmenthol cigarettes at increasing prices. While there was higher demand for one’s preferred versus nonpreferred flavor, no differences emerged between menthol and nonmenthol smokers on “willingness to pay.”^13,31^ An online marketplace study involving 1197 adult menthol smokers (mean age = 41) showed that cigarettes were less likely to be purchased in scenarios simulating either a menthol cigarette ban or a ban on all combustible menthol tobacco products, compared to no ban.^34^ Human laboratory studies with adult menthol smokers further indicate that menthol-flavored alternative tobacco products are popular substitutes for menthol cigarettes, emphasizing the highly reinforcing value of menthol flavoring on tobacco use,^10,33^ although one’s usual menthol cigarette brand remains the most reinforcing of alternatives.^30^ One study among menthol smokers (mean age = 34) that involved a two-week nonmenthol switching phase showed a significant decrease in daily cigarette consumption during the nonmenthol switching phase, particularly among Black participants.^32^ These latter findings suggest a reduction in smoking motivation when a reinforcer (e.g., menthol) is absent.

Little research has determined whether menthol flavoring enhances the reinforcing value of smoking specifically in young adult smokers, despite the popularity of menthol cigarettes in this age group. Further, few studies have examined whether the RRV of menthol (vs. nonmenthol) is greater among subpopulations with high rates of menthol use. This study examined the RRV of menthol versus nonmenthol cigarettes in young adult menthol and nonmenthol smokers using a choice task that assessed willingness to work to earn puffs of menthol and nonmenthol cigarettes. We hypothesized that menthol smokers would “work” harder to earn puffs of their preferred (vs. nonpreferred) cigarette flavor. We also explored interactions of menthol preference with race and ethnicity, as well as sexual orientation on RRV of menthol.

## Materials and Methods

### Participants and Procedures

Young adult menthol and nonmenthol cigarette smokers (age 18–26) were recruited from Oklahoma City, Oklahoma (2020–2023). Eligibility criteria were: (1) current “some days” or “everyday” cigarette smoking; (2) have a strong preference for either menthol or nonmenthol cigarettes (report using one flavor ≥80% of the time when they smoked, consistent with published work^35^); (3) ability to provide informed consent; and (4) willingness to abstain from nicotine and tobacco use ≥ 12 h prior to each of three laboratory visits. We included those with a strong preference for menthol or nonmenthol cigarettes to minimize the risk that some nonmenthol smokers may switch to menthol cigarettes from study participation. Exclusion criteria were: (1) using nicotine replacement therapy (like patch, gum, inhaler, or lozenge); (2) pregnant, planning to become pregnant, or breastfeeding; (3) self-reported certain medical diagnoses (asthma, cardiac distress, lung disease, etc.) that would have precluded successful completion of the study; or (4) inability to adhere to the protocol requirements.

Participants completed three human laboratory smoking sessions following overnight abstinence prior to each session (≥12 h, verified exhaled CO ≤8 ppm). Session 1 measured subjective response to smoking their usual brand cigarette (menthol or nonmenthol cigarette) via an ad-libitum smoking session. In Session 2, participants sampled both menthol and nonmenthol versions (counterbalanced) of a commercially available Camel Crush cigarette (R.J. Reynolds, Winston-Salem),^35^ to become familiar with the cigarette flavors they would be “working” for in the behavioral choice task in Session 3. In Session 2, participants were instructed to take a minimum of 3 and up to 5 puffs of each cigarette flavor (menthol and nonmenthol). There was a 20-minute wash-out period between each cigarette smoked. Using procedures similar to Strasser and colleagues,^35^ commercially available Camel Crush cigarettes were used because they contain a small, menthol-filled capsule that breaks open when squeezed and releases menthol into the cigarette. They were well suited to isolate menthol’s effects because there is no menthol flavor prior to squeezing, there is minimal menthol variation after crushing, and they have similar levels of nicotine, cotinine, and NNK compared to other brands.^35,36^ They are also ideal because they were not popular among young adults, based on an assessment of the Population Assessment of Tobacco and Heath study at the time of funding (Wave 1). Product popularity and preference could unduly influence product perceptions.

In Session 3, participants completed a choice task that assessed willingness to “work” to earn puffs of a menthol and nonmenthol cigarette; adapted from a paradigm developed and tested by Audrian-McGovern^28,29^ to measure tobacco reinforcement. Participants were seated in front of two computer screens and instructed to move a computer mouse so that the cursor hit images (a neutral image alongside an image of a cigarette) on one of the two screens, to earn points toward puffs of each cigarette type. The neutral target (to ensure task attention) was an image of flowers and the nonneutral targets were images of a menthol and nonmenthol cigarette. Cigarette images were intentionally brand neutral, to reduce the potential impact of brand preference and popularity on choice behavior. The menthol cigarette was depicted with a green menthol leaf and the nonmenthol cigarette was depicted with a brown tobacco leaf. Participants read a set of standard instructions, during which they were told to work “for the cigarette you REALLY WANT” (see Supplementary Material for full instructions). Using two concurrent schedules,^37,38^ participants had the choice of working to earn puffs from two different cigarettes (menthol, nonmenthol) and were able to switch from working on one screen to another as often as they desired. Consistent with relative reinforcement paradigms,^23,28,29^ the reinforcement schedule to earn a nonmenthol puff (at each trial) remained fixed over 10 trials, where 25 clicks of the nonmenthol target earned a puff at each trial (maximum possible of 250 clicks over the task for nonmenthol puffs), while The reinforcement schedule to earn a menthol puff increased progressively by 25 clicks at each trial such that 25, 50, 75, 100, 125, 150, 175, 200, 225, and 250 clicks of the menthol cigarette image had to be achieved to earn a menthol puff at each trial (maximum possible of 1375 clicks over the task for menthol puffs). Thus, the task was designed to measure the reinforcing value of menthol cigarette puffs compared to nonmenthol cigarette puffs and thus increasingly more clicks were required to earn a menthol (vs. nonmenthol) puff at each trial. Participants could earn a maximum of 10 puffs regardless of flavor type (1 puff earned at each trial). All participants completed the task until the end (all 10 trials). Cigarette puffs were taken at the end of the task to ensure satiation did not influence responding in subsequent trials. To ensure that participants worked for their preferred reinforcer, rather than the reinforcer requiring less work (e.g., only working for nonmenthol cigarettes to leave the session more quickly), there was a 30-minute wait period in the laboratory once the choice task was completed.^28,29^ This paper focuses on results of Session 3.

Procedures were approved by the IRB (#10581; NCT03953508). Participants were compensated $45 for each study visit. Additional protocol details have been published here.^39^

### Measures

Demographic variables included age, sex, race (White, Black/African American, Asian, American Indian/Alaska Native, Native Hawaiian/Other Pacific Islander, more than one race), ethnicity (Hispanic and non-Hispanic), education (categorized as at least some high school vs. college degree or higher), and sexual orientation (categorized as heterosexual/straight vs. LGBQ [lesbian, gay, bisexual, queer, questioning, don’t know, refused to answer]). Due to small cell sizes, race and ethnicity were categorized as non-Hispanic (NH) White, NH non-White, and Hispanic for the primary analyses. Smoking history included assessments of age of initiation, years smoking, current cigarettes smoked per day, and past 30-day cigarette smoking frequency. Nicotine dependence was assessed with a single item assessing time-to-first cigarette, “*How soon after you wake up do you smoke your first cigarette?*,” *with response options* “within 5 minutes,” “6 to 30 minutes,” “31 to 60 minutes,” and “after 60 minutes.”^40^ This item was dichotomized into smoking within 5 minutes of waking versus smoking 6 minutes or later after waking.

Before beginning the choice task, participants provided ratings of current withdrawal from the 15-item Minnesota Nicotine Withdrawal Scale (MNWS^41^). For this analysis, we used the craving intensity item as a covariate in all analyses to control for any impact it might have had on task responding.

The primary outcome was the RRV of menthol versus nonmenthol cigarettes, which was operationalized as the breakpoint, or the highest trial (out of 10 trials) that a participant worked for a menthol cigarette puff. Higher scores indicated greater willingness to work for a menthol cigarette puff and thus greater RRV for menthol. We also examined two secondary outcomes: total number of clicks for the menthol target/image and total number of clicks for the nonmenthol target/image.

### Statistical Analysis

Analyses focused on *n* = 107 participants who completed the behavioral choice task in Session 3. Descriptive statistics were first used to assess demographic and baseline smoking characteristics of the sample, and bivariate tests were used to examine differences between menthol and nonmenthol smokers on these factors. Next, analysis of covariance tests examined the main effect associations of menthol preference (menthol vs. nonmenthol), race and ethnicity (NH White, NH non-White, Hispanic), and sexual orientation (straight/heterosexual vs. LGBQ) on the primary and secondary choice task outcomes (e.g., breakpoint, number of menthol target clicks, number of nonmenthol target clicks). ANCOVA models controlled for pre-task cigarette craving (i.e., MNWS). Models examining main effects of race, ethnicity, and sexual orientation also controlled for menthol preference to determine their unique influence on the outcomes of interest, above and beyond menthol preference. Baseline cigarettes per day, past 30-day cigarette smoking frequency, and time-to-first cigarette were not significantly related to the outcomes and were not included as covariates. Normality of the outcomes was assessed using tests of kurtosis and skewness (e.g., ±1) and visual inspection of the data. While data were positively skewed, both the log and square root transformations were examined, but neither appreciably altered the distribution of the data or the results of statistical tests. Thus, raw data were analyzed and are presented. Exploratory analyses examined interactions of menthol preference with (1) race and ethnicity and (2) sexual orientation on the outcomes of interest.

## Results

### Sample Characteristics

Table 1 shows the demographic characteristics of the sample and differences between menthol and nonmenthol smokers. Participants were roughly evenly split between menthol (*n* = 54) and nonmenthol (*n* = 53) smokers and were 23.8 years (SD = 2.1) on average. Just over half were female (55.1%), reported at least some college education (51.4%), and identified as straight/heterosexual (54.2%). The majority identified as NH White (62.6%).

**Table 1.** Demographics and Smoking History of Young Adult Menthol and Nonmenthol Smokers

	Menthol preference						*p*
	Nonmenthol		Menthol		Total		
	*n* = 53		*n* = 54		*n* = 107		
Age, M (SD)	24.1	(2.0)	23.4	(2.2)	23.8	(2.1)	ns
Race and ethnicity; *n*, %							
Non-Hispanic (NH) White	32	60.4%	35	64.8%	67	62.6%	ns
NH non-White*	15	28.3%	8	14.8%	23	1.5%	
Hispanic	6	11.3%	11	20.4%	17	15.9%	
Biological sex; *n*, %							
Male	27	50.9%	21	38.9%	48	44.9%	ns
Female	26	49.1%	33	61.1%	59	55.1%	
Highest level of education; *n*, %							
High school or less	30	56.6%	22	40.7%	52	48.6%	ns
Some college or more	23	43.4%	32	59.3%	55	51.4%	
Sexual orientation; *n*, %							
Straight/heterosexual	34	64.2%	24	44.4%	58	54.2%	0.041
LGBQ	19	35.8%	30	55.6%	49	45.8%	
*Smoking history*							
Years smoked; *n*, %							
Less than 5 years	14	26.9%	21	38.9%	35	33.0%	ns
5 + years	38	73.1%	33	61.1%	71	67.0%	
Age started smoking, M (SD)	17.0	(2.9)	17.3	(2.6)	17.2	(2.8)	ns
Cigarettes per day, M (SD)	11.6	(7.7)	8.1	(5.5)	9.8	(6.9)	0.007
P30 day cigarettes smoked, M (SD)	25.4	(8.9)	24.0	(8.6)	24.7	(8.7)	ns
Time-to-first cigarette; *n*, %							
Within 5 minutes of waking	10	18.9%	14	25.9%	24	22.4%	ns
≥6 minutes after waking	43	51.8%	40	48.2%	83	77.6%	
Pre-task cigarette craving, M (SD)	2.68	(1.25)	2.96	(1.13)	2.82	(1.19)	ns

NH non-White category includes: *n* = 3 NH African American/Black, *n* = 7 NH Other (American Indian/Alaska Native, Asian, Native Hawaiian/Pacific Islander), and *n* = 13 NH more than one race. LGBQ = Lesbian, gay, bisexual, queer or questioning.

In terms of smoking history, most reported smoking for five or more years (67%), with a mean age of smoking initiation of 17.2 (SD = 2.8). On average, participants reported smoking 24.7 days of the past 30 days (SD = 8.7) and 9.8 cigarettes per day (SD = 6.9). Roughly a quarter of the sample (22.4%) reported smoking their first cigarette within 5 minutes of waking, indicating high levels of nicotine dependence. No significant differences were found between menthol and nonmenthol smokers on the majority of demographic and smoking variables, with the exception that menthol smokers reported smoking fewer cigarettes per day than nonmenthol smokers (8.1 vs. 11.6, *p* < .01) and were more likely to identify as LGBQ (55.6% vs. 35.8%, *p* < .05). Menthol and nonmenthol smokers did not differ on pre-choice task ratings of cigarette craving (*p* = .22).

### Effects of Menthol Preference on Choice Task Outcomes

Choice task completion time (in minutes) was higher for menthol versus nonmenthol smokers (*M* = 45.76 vs. 15.04 minutes), *p* < .001). Main effects models showed that menthol preference was associated with all choice task outcomes (Table 2; all *p*’s < .05), controlling for pre-task craving. Menthol smokers worked harder to earn menthol versus nonmenthol cigarette puffs (breakpoint: 9.17), clicking the menthol target ~1,236 times and the nonmenthol target ~24 times. Nonmenthol smokers had an average breakpoint of 0.83, and clicked the nonmenthol target 231 times and the menthol target 105 times.

**Table 2.** Adjusted Means, Standard Errors, and Analysis of Covariance (ANCOVA) Results of the Main Effects of Menthol Cigarette Preference, Race and Ethnicity, and Sexual Orientation on Choice Task Outcomes

	Breakpoint	# of clicks for a nonmenthol cigarette puff (max possible 250)	# of clicks for a menthol cigarette puff (max possible 1375)
Main effect factor	Mean (SE)	Mean (SE)	Mean (SE)
Menthol preference			
Menthol	9.17 (0.32)	24.15 (8.27)	1235.82 (47.22)
Nonmenthol	0.83 (0.31)	231.40 (8.11)	105.12 (46.31)
* F*(df)	334.10*** (1,104)	317.57*** (1,104)	290.17*** (1,104)
* *η_p_^2^	0.76	0.75	0.74
Race and ethnicity^¶¶^			
NH White	4.83 (0.28)^a^	131.33 (7.19)^a^	649.17 (40.85)^a^
NH non-White	4.43 (0.47)^a^	141.39 (12.26)^a^	585.92 (69.61)^a^
Hispanic	6.49 (0.56)^b^	94.03 (14.48)^b^	876.86 (82.23)^b^
* F*(df)	4.37* (2,103)	3.44* (2,103)	4.41* (2,103)
* * η_p_^2^	0.06	0.06	0.07
Sexual orientation^¶¶^			
Straight/heterosexual	4.58 (0.30)	138.79 (7.85)	613.10 (44.95)
LGBQ	5.48 (0.33)	115.02 (8.46)	736.88 (48.44)
* F*(df)	3.80^**±**^ (1,104)	4.41** (1,104)	3.42^**±**^ (1,104)
* * η_p_^2^	0.03	0.04	0.03

^±^ *p* < .10, *p < .05, **p < .01, ***p < .001. Items with different superscripts in the same column differ significantly at *p* < .05.

All models controlled for pre-task cigarette craving (*n* = 3 people were missing responses on this item).

^¶¶^ANCOVA models also controlled for menthol preference.

### Effects of Race and Ethnicity on Choice Task Outcomes

Controlling for pre-task craving and menthol preference, race, and ethnicity were associated with all choice task outcomes (Table 2). In post-hoc tests, breakpoint for menthol was higher among Hispanic smokers (breakpoint = 6.49) compared to NH White (breakpoint = 4.83) and to NH non-White smokers (breakpoint = 4.43) (*p*’s < .05).

Hispanic smokers clicked, on average, more times for the menthol target (876 clicks) compared to both NH White (649 clicks) and to NH non-White smokers (585 clicks) (*p*’s < .05).

Hispanic smokers clicked, on average, fewer times for the nonmenthol target (94 clicks) compared to both NH White (131 clicks) and NH non-White respondents (141 clicks) (*p*’s < .05).

NH White and NH non-White respondents did not differ on any choice task outcomes (breakpoint for menthol, average number of menthol target clicks, average number of nonmenthol target clicks).

### Effects of Sexual Orientation on Choice Task Outcomes

LGBQ (vs. straight/heterosexual) respondents clicked fewer times for the nonmenthol target (115 vs. 138, *p* < .01), but no differences emerged for breakpoint and number of menthol clicks (see Table 2).

### Exploratory Analyses: Interactions of Menthol Preference With Race and Ethnicity, and Sexual Orientation

**Figure 1:**
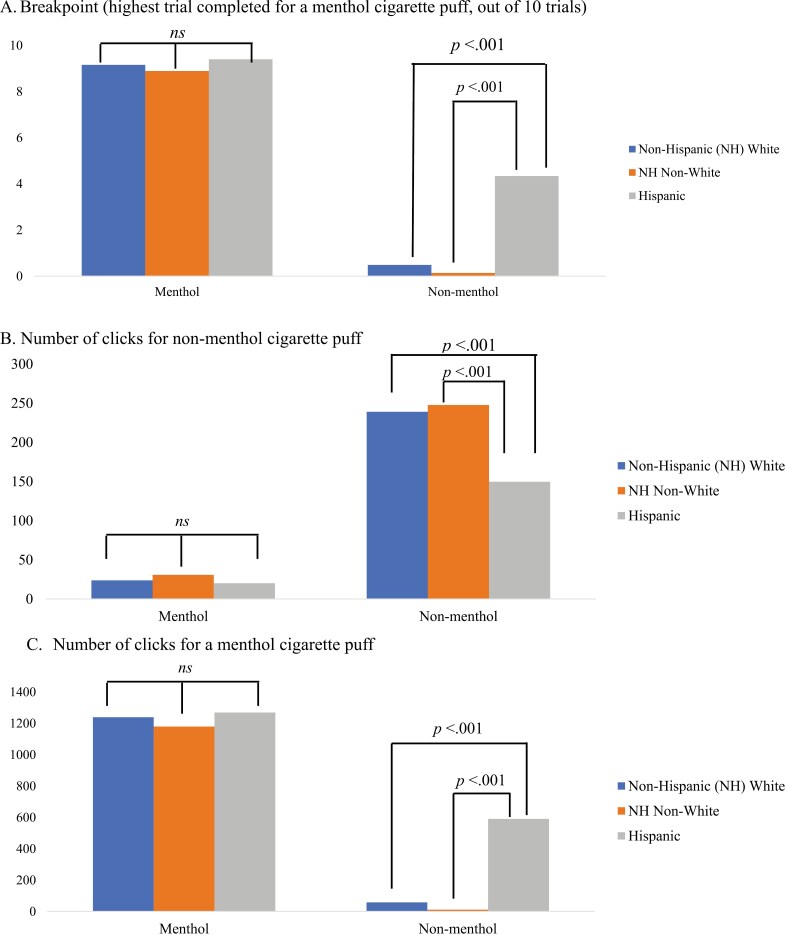
(A–C) Bar graphs displaying differences in behavioral economic choice task outcomes by menthol preference and race and ethnicity [non-Hispanic (NH) White, NH non-White, Hispanic]. Data represent adjusted means controlling for pre-task cigarette craving. For breakpoint, higher trials completed for a menthol cigarette puff indicate greater willingness to “work” to earn a cigarette puff. Menthol smokers were not significantly (ns) different on all choice task outcomes across race and ethnicity, while nonmenthol smokers were significantly different. Sample sizes: *n* = 32 nonmenthol NH White, *n* = 15 nonmenthol NH non-White, *n* = 6 nonmenthol Hispanic, *n* = 33 menthol NH White, *n* = 8 menthol NH non-White, *n* = 10 menthol Hispanic.

Figure 1 shows significant interactions of menthol preference with race and ethnicity on the study outcomes. Table S1 shows pairwise comparisons with post-hoc test results. The plot of the means shows an effect of race and ethnicity on all outcomes specifically among the nonmenthol smokers (*p*’s < .001), but not among the menthol smokers (*p*’s > .05). Specifically, Hispanic nonmenthol smokers (*n* = 6) had higher breakpoints and clicked the menthol target more times compared to the NH White smokers (*n* = 32) and NH non-White smokers (*n* = 15) (*p*’s < .001). Similarly, Hispanic nonmenthol smokers, compared to NH White and NH non-White nonmenthol smokers, clicked the nonmenthol target fewer times (*p*’s < .001). No interactions of menthol preference with sexual orientation emerged.

## Discussion

Findings bolster evidence of previous studies showing a high reinforcing value of menthol (vs. nonmenthol), a key indicator of cigarette addiction potential. As hypothesized, young adult menthol smokers expended more effort to earn cigarette puffs of their preferred versus nonpreferred flavor. Young adults without such a preference were not willing to expend similar effort. These young adults likely find nonmenthol cigarettes more reinforcing then menthol cigarettes, though we did not directly assess this in the current study. Importantly, we note that menthol and nonmenthol smokers did not differ on a range of demographic and tobacco use characteristics that could have influenced choice task responding, further suggesting that the high reinforcing value of menthol cigarettes might motivate some groups of young adult smokers to work exceedingly hard to earn puffs of this product (when an alternative is present). Furthermore, menthol (vs. nonmenthol) smokers smoked fewer cigarettes per day but had similar levels of nicotine dependence, suggesting that the high reinforcing value of menthol in cigarettes may be an early indicator of subsequent greater nicotine dependence, leading to reduced smoking cessation success.^4,6,13^ Menthol have been shown to impact nicotine metabolism and absorption,^42–44^ which might also explain why menthol smokers consumed fewer cigarettes per day but had similar levels of nicotine dependence.

Beyond the effects of menthol cigarette preference, demographic factors linked to greater menthol use (e.g., race, ethnicity, and sexual orientation) were also associated with choice task responding that favored menthol cigarette puffs. In main effects models controlling for both menthol preference and pre-task craving, Hispanic smokers, compared to NH White and NH non-White smokers had higher breakpoints, clicked the menthol target more times and the nonmenthol target fewer times. Interestingly, in exploratory moderation analyses, willingness to work for menthol cigarette puffs was higher among Hispanic nonmenthol smokers compared to NH White and NH non-White nonmenthol smokers. Further, the number of clicks for the nonmenthol target was lower among Hispanic nonmenthol smokers compared to these two other groups. Among the menthol smokers, all three race and ethnicity groups showed an equal degree of effort for menthol cigarette puffs. Although sample sizes were small and require replication in a larger sample, these exploratory analyses raise the possibility that even nonmenthol Hispanic smokers have some affinity for menthol, a possible by-product of tobacco company targeting,^14,19,45^ exposure to peers who use menthol (given high rates of menthol use among Hispanic smokers), and/or cultural affiliation. For example, menthol cigarette preference may arise as an “expression of belonging” to one’s cultural group (pp.S33),^46^ and could align with cultural beliefs about the medicinal benefits of menthol.

A greater proportion of LGBQ (vs. heterosexual) young adults in our study identified as a menthol smoker, consistent with other work.^1^ The proportion of LGBQ participants in our sample was higher than U.S. epidemiological estimates, allowing us to specifically examine this important high-risk subgroup of smokers. Although LGBQ participants clicked the nonmenthol target fewer times than straight/heterosexual participants, willingness to work for menthol puffs (e.g., breakpoint) was equal across LGBQ and straight/heterosexual participants. This latter finding was somewhat surprising, given the literature showing that menthol smoking is higher among sexual minority smokers compared to heterosexual smokers.^1,47^ Perhaps other factors linked to tobacco use among LGBQ young adults, like minority stress experiences or worse mental health, interact more strongly with LGBQ identity in the prediction of smoking behavior than menthol preference. The absence of an association between sexual orientation and greater willingness to work for menthol cigarette puffs could also be because sexual minority (vs. heterosexual) individuals tend to favor a range of flavored tobacco products beyond menthol cigarettes.^48^ This may explain, in part, why LGBQ participants clicked fewer times than straight/heterosexual participants for the nonmenthol cigarette. More studies are needed to determine the mechanisms linking race, ethnicity, and sexual orientation to menthol cigarette preference.

This study extends the literature. This is one of the first studies to examine RRV of menthol versus nonmenthol cigarettes specifically in young adult smokers, and to explore these effects across Black, Hispanic, and sexual minority individuals. We focused on young adult smokers, a group that is relatively new to smoking compared to older adult smokers who have historically been the focus of experimental studies assessing menthol’s appeal. This reduces the potential confound of longstanding flavor preference on behavioral responses. The choice task provides valuable evidence of the addiction potential of menthol, as participants who worked for menthol puffs delivered exceedingly more effort to obtain those puffs, even when an alternative (nonmenthol cigarettes) was present. This aligns with real-world experiences, in which consumers are exposed simultaneously to multiple available tobacco products and can “control” their cigarette consumption by changing their use patterns (e.g., switching to nonmenthol products), such as if menthol cigarettes were to be restricted or heavily taxed. Other findings also show a higher reinforcing value for menthol (vs. nonmenthol) cigarettes even among groups of participants who did not have a strong preference for menthol.

This study had several limitations. Due to small sample sizes, we could not compare menthol’s RRV among Black participants to that of other racial and ethnic groups in the sample. Future research with larger samples is necessary to more conclusively evaluate menthol preferences among minoritized groups of emerging adults. Participants were enrolled specifically for their preference for menthol or nonmenthol cigarettes, which could have driven disparities in choice task responding. However, it is unlikely our sample differed from the typical young adult menthol and nonmenthol smoker as switching from menthol to nonmenthol cigarette smoking, or vice versa, is uncommon, and most smokers use the flavor type they initiated with.^2,49,50^ We cannot completely disentangle the influence of environmental (e.g., marketing) and biological (e.g., taste sensitivity) factors impacting menthol preference with the study outcomes.^21^ We focused specifically on cigarettes, though the reinforcing value of other menthol-flavored nicotine/tobacco products should be examined. A variety of contextual factors outside of drug properties could influence menthol’s reinforcement, including comorbidity and reduced access to other ways of experiencing reward. We note, however, there were no group differences in this study on many nondrug factors (demographics).

## Conclusions

This study answers an important question about whether, in younger users, menthol increases the reinforcing properties of cigarette smoking. This is important given the highly appealing nature of menthol cigarettes as a “starter product”^2,5,12^ and tobacco company literature suggesting high curiosity and appeal for menthol cigarettes in young adults.^22^ The findings suggest that, even among young adults with a relatively brief smoking history, the effects of menthol on choice behavior are robust. The fact that menthol is relatively more reinforcing than nonmenthol among some ethnically marginally groups without a menthol preference further highlights the impact that menthol may have on smoking persistence and the role a menthol ban may have on reducing cigarette smoking in the next generation of smokers. Study findings can help inform the development of smoking cessation programs specific to young adult menthol smokers as well as prevention messaging designed to reduce menthol cigarette appeal, particularly targeting vulnerable groups.

## Supplementary material

Supplementary material is available at *Nicotine and Tobacco Research* online.

ntae186_suppl_Supplementary_Material

ntae186_suppl_Supplementary_Table_S1

## Data Availability

De-identified data supporting the findings of this study can be shared upon reasonable request from the corresponding author, with the permission of the University of Oklahoma Health Sciences Center. The data are not publicly available due to privacy restrictions.
