# Supplementary material for: The Relative Reinforcing Value of Menthol Among Young Adult Cigarette Smokers: Results From a Behavioral Choice Task
Source: Nicotine Tob Res. 2024 Sep 18;27(7):1177–85. doi: 10.1093/ntr/ntae186 (PMC12187066; doi:10.1093/ntr/ntae186)
Supplement: ntae186_suppl_Supplementary_Material [file ntae186_suppl_supplementary_material.docx]

Supplemental material: Choice task instructions shown to participants

*In this part of the session you will play a simple computer task for two rewards: puffs of a non-menthol cigarette or a menthol cigarette. The task has 10 rounds so you will have a chance to earn 10 cigarette puffs. Press any key to continue.*

*First, we want to make you familiar with the task. On one side of the screen you can collect menthol puffs, and the other you can collect non-menthol puffs. You may alternate sides by moving the cursor back and forth and clicking on the side of the screen you want to use. Press any key to continue.*

*You collect puffs of a non-menthol or a menthol cigarette by clicking on the picture. The non-menthol cigarette looks like this [insert image of non-menthol cigarette]. The menthol cigarette looks like this [insert image of menthol cigarette].However, if you click on the flowers you will be delayed and must click the flowers again to return to your work [insert image of flowers]. Therefore you must move the mouse carefully. Here is a practice round for you.*

*[Following practice round] “Remember, you may switch back and forth between earning menthol and non-menthol whenever you want. There are 10 rounds, giving a chance to earn 10 cigarettes puffs total. On each round, you can earn a puff of a menthol or non-menthol cigarette, depending on which cigarette you are playing for. Play for the cigarette that you REALLY WANT.*

*Press any key to continue.*
