# Supplementary material for: The Relative Reinforcing Value of Menthol Among Young Adult Cigarette Smokers: Results From a Behavioral Choice Task
Source: Nicotine Tob Res. 2024 Sep 18;27(7):1177–85. doi: 10.1093/ntr/ntae186 (PMC12187066; doi:10.1093/ntr/ntae186)
Supplement: ntae186_suppl_Supplementary_Table_S1 [file ntae186_suppl_supplementary_table_s1.docx]

*Supplemental Table 1*. Pairwise comparisons test results (adjusted means and standard errors) of the interactions of menthol preference and race and ethnicity on choice task outcomes.

|  | **Race and Ethnicity** | | |  |
| --- | --- | --- | --- | --- |
| Choice Task Outcome | Non-Hispanic (NH) White | NH Non-White | Hispanic |  |
| **Breakpoint** | Mean (SE) | Mean (SE) | Mean (SE) | Simple Effects: *F*  *df*(2, 97) |
| Menthol preference | 9.16 (0.38) ^a^ | 8.89 (0.76) ^a^ | 9.40 (0.68) ^a^ | 0.12 |
| Non-menthol preference | 0.49 (0.38)^a^ | 0.14 (0.56)^a^ | 4.35 (0.88)^b^ | 9.05*** |
| Simple Effects: *F*  *df*(1,97) | 257.94*** | 85.37*** | 20.52*** |  |
| **Number of clicks for a non-menthol cigarette** |  |  |  | Simple Effects: *F*  *df*(2, 97) |
| Menthol preference | 23.81 (9.8) ^a^ | 30.80 (19.89) ^a^ | 20.11 (17.70) ^a^ | 0.08 |
| Non-menthol preference | 239.01 (9.89)^a^ | 247.74 (14.58)^a^ | 149.66 (22.89)^b^ | 7.33** |
| Simple Effects: *F*  *df*(1,97) | 237.15*** | 78.44*** | 20.85*** |  |
| **Number of clicks for a menthol cigarette** |  |  |  | Simple Effects: *F*  *df*(2, 97) |
| Menthol preference | 1239.18 (56.01) ^a^ | 1179.40 (112.729) ^a^ | 1269.00 (100.32) ^a^ | 0.18 |
| Non-menthol preference | 58.24 (56.05)^a^ | 11.50 (82.63)^a^ | 590.71 (129.72)^b^ | 8.03*** |
| Simple Effects: *F*  *df*(1,97) | 222.40*** | 70.79*** | 17.14*** |  |

Note. ^±^  *p* < .10 * *p* < .05, ** *p* < .01, *** *p* < .001

Models controlled pre-task cigarette craving. Items with different superscripts in the same row differ significantly at *p*<.05.
